# Supplementary material for: Multi‐dataset identification of innovative feature genes and molecular mechanisms in keratoconus
Source: J Cell Mol Med. 2024 Sep 19;28(18):e70079. doi: 10.1111/jcmm.70079 (PMC11412914; doi:10.1111/jcmm.70079)
Supplement: Supplementary file 2 — Material S2. [file JCMM-28-e70079-s002.zip › 7.GSEA analysis/1.ARL11/KEGG_VIBRIO_CHOLERAE_INFECTION.html]

Details for gene set KEGG\_VIBRIO\_CHOLERAE\_INFECTION[GSEA]

|  || Dataset | ARL11.group.cls#h\_versus\_l |
| Phenotype | group.cls#h\_versus\_l |
| Upregulated in class | h |
| GeneSet | KEGG\_VIBRIO\_CHOLERAE\_INFECTION |
| Enrichment Score (ES) | 0.22926544 |
| Normalized Enrichment Score (NES) | 0.958941 |
| Nominal p-value | 0.532 |
| FDR q-value | 1.0 |
| FWER p-Value | 1.0 |
Table: GSEA Results Summary

  

Fig 1: Enrichment plot: KEGG\_VIBRIO\_CHOLERAE\_INFECTION      
 Profile of the Running ES Score & Positions of GeneSet Members on the Rank Ordered List

  

| SYMBOL | TITLE | RANK IN GENE LIST | RANK METRIC SCORE | RUNNING ES | CORE ENRICHMENT || 1 | SEC61A2 | na | 2129 | 0.287 | 0.0153 | Yes |
| 2 | KCNQ1 | na | 2965 | 0.242 | 0.0558 | Yes |
| 3 | ATP6V0A4 | na | 4084 | 0.198 | 0.0764 | Yes |
| 4 | PLCG1 | na | 4334 | 0.189 | 0.1197 | Yes |
| 5 | ADCY3 | na | 5276 | 0.158 | 0.1347 | Yes |
| 6 | ADCY9 | na | 5439 | 0.153 | 0.1710 | Yes |
| 7 | PLCG2 | na | 6096 | 0.135 | 0.1880 | Yes |
| 8 | ATP6V0D1 | na | 6786 | 0.117 | 0.1993 | Yes |
| 9 | SLC12A2 | na | 6821 | 0.116 | 0.2293 | Yes |
| 10 | MUC2 | na | 9506 | 0.067 | 0.1698 | No |
| 11 | PRKX | na | 9610 | 0.066 | 0.1844 | No |
| 12 | PRKCA | na | 10617 | 0.052 | 0.1693 | No |
| 13 | ATP6V1B2 | na | 12446 | 0.032 | 0.1252 | No |
| 14 | ATP6AP1 | na | 12793 | 0.029 | 0.1229 | No |
| 15 | KDELR1 | na | 12806 | 0.029 | 0.1302 | No |
| 16 | ACTB | na | 12961 | 0.027 | 0.1330 | No |
| 17 | ATP6V1E2 | na | 13257 | 0.025 | 0.1311 | No |
| 18 | ATP6V1B1 | na | 14360 | 0.016 | 0.1037 | No |
| 19 | ATP6V0E2 | na | 14410 | 0.016 | 0.1065 | No |
| 20 | ATP6V1G3 | na | 14530 | 0.015 | 0.1070 | No |
| 21 | ATP6V0A1 | na | 15062 | 0.011 | 0.0945 | No |
| 22 | ATP6V0B | na | 15548 | 0.007 | 0.0825 | No |
| 23 | TJP1 | na | 15790 | 0.005 | 0.0769 | No |
| 24 | GNAS | na | 16257 | 0.002 | 0.0640 | No |
| 25 | SEC61B | na | 16352 | 0.001 | 0.0616 | No |
| 26 | ATP6V1F | na | 17512 | -0.007 | 0.0300 | No |
| 27 | PRKCG | na | 17545 | -0.007 | 0.0310 | No |
| 28 | ATP6V0C | na | 17696 | -0.008 | 0.0289 | No |
| 29 | ATP6V1C1 | na | 18651 | -0.015 | 0.0052 | No |
| 30 | PRKACB | na | 18969 | -0.017 | 0.0006 | No |
| 31 | ATP6V0E1 | na | 19061 | -0.017 | 0.0026 | No |
| 32 | TJP2 | na | 19172 | -0.018 | 0.0042 | No |
| 33 | ATP6V1D | na | 20147 | -0.025 | -0.0173 | No |
| 34 | ACTG1 | na | 20219 | -0.025 | -0.0126 | No |
| 35 | ATP6V1G2 | na | 20600 | -0.028 | -0.0160 | No |
| 36 | ATP6V1A | na | 20723 | -0.029 | -0.0117 | No |
| 37 | ATP6V1G1 | na | 21288 | -0.033 | -0.0191 | No |
| 38 | SEC61G | na | 22550 | -0.046 | -0.0433 | No |
| 39 | SEC61A1 | na | 22654 | -0.047 | -0.0337 | No |
| 40 | PRKACA | na | 23665 | -0.058 | -0.0474 | No |
| 41 | ATP6V1E1 | na | 23880 | -0.061 | -0.0374 | No |
| 42 | ATP6V0D2 | na | 24169 | -0.065 | -0.0284 | No |
| 43 | KDELR2 | na | 24186 | -0.065 | -0.0116 | No |
| 44 | PDIA4 | na | 24357 | -0.067 | 0.0014 | No |
| 45 | ARF1 | na | 24865 | -0.074 | 0.0066 | No |
| 46 | ATP6V1C2 | na | 25402 | -0.081 | 0.0129 | No |
| 47 | PRKACG | na | 25752 | -0.087 | 0.0259 | No |
| 48 | ATP6V1H | na | 25843 | -0.088 | 0.0468 | No |
| 49 | CFTR | na | 27887 | -0.128 | 0.0220 | No |
| 50 | KDELR3 | na | 31931 | -0.257 | -0.0259 | No |
| 51 | PRKCB | na | 33863 | -0.399 | 0.0249 | No |
Table: GSEA details [plain text format]

  

Fig 2: KEGG\_VIBRIO\_CHOLERAE\_INFECTION      
 Blue-Pink O' Gram in the Space of the Analyzed GeneSet

  

Fig 3: KEGG\_VIBRIO\_CHOLERAE\_INFECTION: Random ES distribution      
 Gene set null distribution of ES for **KEGG\_VIBRIO\_CHOLERAE\_INFECTION**
